# Tobacco Product Use Among Middle and High School Students — National Youth Tobacco Survey, United States, 2024

**DOI:** 10.15585/mmwr.mm7341a2

**Published:** 2024-10-17

**Authors:** Ahmed Jamal, Eunice Park-Lee, Jan Birdsey, Andrenita West, Monica Cornelius, Maria R. Cooper, Hannah Cowan, Jia Wang, Michael D. Sawdey, Karen A. Cullen, Livia Navon

**Affiliations:** ^1^Office on Smoking and Health, National Center for Chronic Disease Prevention and Health Promotion, CDC; ^2^Center for Tobacco Products, Food and Drug Administration, Silver Spring, Maryland.

**Summary**

**What is already known about this topic?**

Use of tobacco products in any form is unsafe; most tobacco product use begins in adolescence.

**What is added by this report?**

From 2023 to 2024, current (previous 30-day) use of any tobacco product declined among high school students from 12.6% to 10.1%, largely driven by the decline in high school e-cigarette use (from 10.0% to 7.8%). During 2024, e-cigarettes remained the most commonly used tobacco product among U.S. youths; nicotine pouches were the second most commonly used tobacco product.

**What are the implications for public health?**

Tobacco use among youths has continued to decline; however, comprehensive and sustained implementation of evidence-based tobacco control strategies, including tobacco product regulation and enforcement, is needed to prevent and reduce all forms of youth tobacco product use.

## Abstract

Use of tobacco products in any form is unsafe, and nearly all tobacco product use begins during adolescence. CDC and the Food and Drug Administration (FDA) analyzed data from the 2024 National Youth Tobacco Survey to determine tobacco product use among U.S. middle school (grades 6–8) and high school (grades 9–12) students. In 2024, current (previous 30-day) use of any tobacco product was reported by 10.1% of high school students (representing 1.58 million students) and 5.4% of middle school students (representing 640,000 students). Among all students, e-cigarettes were the most commonly reported tobacco product currently used (5.9%), followed by nicotine pouches (1.8%), cigarettes (1.4%), cigars (1.2%), smokeless tobacco (1.2%), other oral nicotine products (1.2%), heated tobacco products (0.8%), hookahs (0.7%), and pipe tobacco (0.5%). During 2023–2024, among all students, the estimated number who reported current use of any tobacco product decreased from 2.80 to 2.25 million students; e-cigarette use decreased (from 2.13 to 1.63 million students); and hookah use decreased (from 290,000 to 190,000 students). Among high school students, current use of any tobacco product decreased from 12.6% to 10.1% of students, and e-cigarette use decreased from 10.0% to 7.8%. Among middle school students, no statistically significant changes occurred. Evidence-based strategies can help prevent initiation and promote cessation of tobacco product use among U.S. youths.

## Introduction

Use of tobacco* products in any form is unsafe, and nearly all tobacco product use begins during adolescence (*1*). This report presents findings from the 2024 National Youth Tobacco Survey (NYTS) and describes ever use and current use of nine tobacco product types and changes in use among U.S. middle and high school students (youths) from 2023 to 2024. Detailed NYTS estimates of e-cigarette and nicotine pouch use were recently published (*2*); this report provides information on use of all forms of tobacco products and includes estimates by school level, sex, and race and ethnicity.

## Methods

### Data Source and Collection

NYTS is a cross-sectional, voluntary, school-based, self-administered, Internet survey of U.S. middle school (grades 6–8) and high school (grades 9–12) students. A stratified, three-stage cluster sampling procedure was used to generate a nationally representative sample of U.S. students attending private or public middle and high schools. Data were collected during January 22–May 22, 2024; 29,861 students from 283 schools participated, with an overall response rate of 33.4%.

### Data Analysis

National weighted prevalence estimates, 95% CIs, and population totals^†^ were calculated for ever use (i.e., ever having used, even once or twice) and current use (i.e., use on ≥1 day during the previous 30 days) of nine tobacco products^§^ (e-cigarettes, nicotine pouches,^¶^ cigarettes, cigars, smokeless tobacco, other oral nicotine products, heated tobacco products,** hookahs, and pipe tobacco) by student characteristics. Three composite use measures were also reported: 1) any tobacco product use,^††^ 2) any combustible tobacco product use,^§§^ and 3) multiple tobacco product use.^¶¶^ Changes in current use prevalence since 2023 were assessed for statistical significance using *t*-tests; 2023 NYTS methods and estimates have been published previously (*3*). P-values <0.05 were considered statistically significant. Analyses were conducted using SAS-callable SUDAAN software (version 11.0.4; Research Triangle Institute). Estimates with an unweighted denominator <50 or a relative SE >30% were suppressed. This activity was reviewed by CDC, deemed not research, and was conducted consistent with applicable federal law and CDC policy.***

## Results

### Characteristics of Students Who Have Ever Used or Currently Use Tobacco Products

In 2024, 19.0% of U.S. middle and high school students (representing 5.28 million students) reported ever having used any tobacco product (Table 1); 8.1% (representing 2.25 million students) reported current use of any tobacco product (Table 2). Current use of any tobacco product was reported by 8.5% of male, 7.7% of female, 16.3% of non-Hispanic American Indian or Alaska Native (AI/AN), 10.0% of non-Hispanic Black or African American (Black), 9.0% of non-Hispanic multiracial (multiracial), 8.4% of Hispanic or Latino (Hispanic), 7.8% of non-Hispanic White (White), and 3.3% of non-Hispanic Asian (Asian) students.^†††^ Current use of any combustible tobacco product was reported by 6.3% of AI/AN, 4.1% of Black, 3.9% of multiracial, 2.9% of Hispanic, and 2.4% of White students. Multiple tobacco product use was reported by 6.9% of AI/AN, 3.8% of multiracial, 3.3% of Black, 3.1% of Hispanic, 3.0% of White, and 1.1% of Asian students. Among students who had ever used a tobacco product, 42.9% reported current use.

**TABLE 1:** Percentage of middle and high school students who reported ever using tobacco products,* overall and by school level, product, sex, and race and ethnicity — National Youth Tobacco Survey, United States, 2024

Tobacco product	% (95% CI)										Total estimated no.^§^
	Sex		Race and ethnicity^†^							Total	
	Female	Male	AI/AN	Asian	Black or African American	NH/PI	White	Hispanic or Latino	Multiracial		
**Overall**											
Any tobacco product^¶^	19.0 (17.5–20.7)	19.0 (17.6–20.6)	31.7 (26.7–37.1)	9.3 (7.2–12.0)	21.6 (19.0–24.6)	17.8 (11.4–26.8)	18.6 (16.7–20.7)	19.7 (18.2–21.3)	22.2 (19.2–25.6)	**19.0 (17.7–20.5)**	**5,280,000**
E-cigarettes	14.8 (13.6–16.2)	13.2 (12.0–14.6)	22.5 (18.0–27.7)	5.7 (4.2–7.8)	14.3 (12.3–16.7)	13.8 (8.0–22.8)	14.1 (12.4–16.0)	15.0 (13.7–16.3)	16.2 (13.7–19.1)	**14.0 (12.9–15.2)**	**3,870,000**
Cigarettes	5.3 (4.6–6.0)	6.3 (5.5–7.2)	11.2 (8.2–15.0)	2.6 (1.9–3.6)	4.4 (3.7–5.3)	—**	6.5 (5.5–7.6)	5.7 (5.0–6.5)	7.5 (5.6–9.8)	**5.8 (5.1–6.5)**	**1,570,000**
Cigars^††^	2.6 (2.2–3.0)	5.1 (4.4–5.8)	6.1 (4.2–8.9)	—	4.6 (3.5–6.1)	—	4.1 (3.5–4.8)	3.7 (3.1–4.3)	4.4 (2.9–6.7)	**3.9 (3.5–4.3)**	**1,050,000**
Nicotine pouches	1.9 (1.6–2.2)	5.0 (4.3–5.9)	9.1 (6.5–12.5)	1.2 (0.7–2.0)	1.7 (1.2–2.4)	—	4.4 (3.7–5.3)	3.0 (2.5–3.5)	3.8 (2.7–5.4)	**3.5 (3.0–4.0)**	**890,000**
Other oral nicotine products^††^	2.6 (2.3–2.9)	3.7 (3.2–4.2)	6.0 (3.8–9.4)	1.3 (0.8–2.2)	2.1 (1.5–3.0)	—	3.4 (2.8–4.1)	3.4 (3.0–3.8)	3.5 (2.6–4.8)	**3.1 (2.8–3.5)**	**840,000**
Smokeless tobacco^††^	1.8 (1.5–2.2)	4.0 (3.3–4.7)	7.7 (5.5–10.7)	1.1 (0.7–1.8)	1.7 (1.2–2.4)	—	3.6 (2.9–4.4)	2.6 (2.2–3.0)	3.4 (2.4–4.9)	**2.9 (2.5–3.4)**	**790,000**
Hookahs	2.6 (2.1–3.1)	2.3 (1.9–2.8)	4.4 (2.7–7.3)	1.5 (1.0–2.5)	4.5 (3.7–5.6)	—	1.5 (1.2–2.0)	2.8 (2.2–3.6)	2.8 (1.9–4.2)	**2.4 (2.1–2.8)**	**650,000**
Heated tobacco products	1.4 (1.1–1.7)	1.7 (1.4–2.1)	3.9 (2.4–6.4)	—	1.9 (1.3–2.7)	—	1.2 (0.9–1.6)	2.1 (1.8–2.4)	1.1 (0.7–1.9)	**1.6 (1.3–1.8)**	**390,000**
Pipe tobacco	1.2 (1.0–1.5)	1.6 (1.4–2.0)	4.8 (3.0–7.5)	—	1.3 (0.9–1.9)	—	1.4 (1.1–1.6)	1.7 (1.4–2.0)	2.0 (1.3–2.9)	**1.5 (1.3–1.7)**	**390,000**
Any combustible tobacco product^§§^	8.5 (7.5–9.5)	10.4 (9.4–11.4)	16.4 (13.0–20.4)	4.1 (3.1–5.5)	11.6 (10.0–13.4)	8.0 (4.5–14.0)	9.3 (8.1–10.7)	9.2 (8.2–10.4)	12.1 (9.8–14.8)	**9.4 (8.6–10.3)**	**2,580,000**
Multiple tobacco products^¶¶^	7.6 (6.8–8.5)	9.5 (8.5–10.7)	17.2 (13.6–21.5)	3.2 (2.3–4.3)	8.3 (6.9–9.9)	—	8.9 (7.7–10.4)	8.8 (7.8–9.9)	10.0 (8.2–12.2)	**8.6 (7.8–9.5)**	**2,380,000**
**High school students (grades 9–12)**											
Any tobacco product^¶^	23.2 (21.2–25.3)	23.9 (21.6–26.4)	37.1 (30.3–44.6)	10.8 (8.1–14.1)	24.2 (20.4–28.5)	25.7 (15.0–40.3)	24.6 (22.2–27.2)	23.5 (21.1–26.0)	25.8 (21.5–30.5)	**23.6 (21.6–25.6)**	**3,700,000**
E-cigarettes	18.8 (17.0–20.8)	17.4 (15.3–19.6)	27.0 (20.8–34.2)	7.0 (5.0–9.7)	17.0 (14.0–20.4)	21.7 (12.1–35.8)	19.3 (16.9–21.9)	18.4 (16.5–20.5)	20.1 (16.1–24.8)	**18.1 (16.3–20.0)**	**2,840,000**
Cigarettes	6.4 (5.5–7.5)	8.3 (7.1–9.6)	15.7 (10.9–22.1)	2.9 (2.0–4.1)	4.3 (3.4–5.6)	—	8.9 (7.5–10.5)	6.8 (5.8–8.0)	9.2 (6.5–12.9)	**7.4 (6.4–8.4)**	**1,130,000**
Cigars^††^	3.1 (2.6–3.7)	7.3 (6.3–8.5)	7.2 (4.4–11.6)	—	5.6 (3.9–8.0)	—	6.1 (5.2–7.2)	4.4 (3.6–5.5)	6.4 (4.0–10.1)	**5.3 (4.6–6.0)**	**800,000**
Nicotine pouches	2.2 (1.8–2.7)	7.2 (5.9–8.6)	12.3 (8.6–17.3)	—	1.9 (1.2–2.8)	—	6.5 (5.4–7.8)	3.6 (2.9–4.5)	4.9 (3.2–7.5)	**4.7 (4.0–5.6)**	**680,000**
Other oral nicotine products^††^	2.8 (2.4–3.3)	4.8 (4.0–5.6)	8.5 (5.1–14.0)	—	2.3 (1.4–3.6)	—	4.5 (3.8–5.5)	3.7 (3.1–4.5)	3.8 (2.4–5.8)	**3.8 (3.3–4.4)**	**570,000**
Smokeless tobacco^††^	1.7 (1.3–2.2)	4.9 (4.0–6.1)	9.4 (6.1–14.3)	—	1.6 (1.0–2.8)	—	4.5 (3.5–5.6)	2.7 (2.1–3.4)	3.7 (2.3–5.9)	**3.4 (2.8–4.1)**	**510,000**
Hookahs	3.2 (2.5–4.0)	3.1 (2.4–3.9)	—	—	5.4 (4.1–7.0)	—	2.1 (1.6–2.9)	3.5 (2.5–4.9)	3.7 (2.3–5.8)	**3.1 (2.6–3.8)**	**470,000**
Heated tobacco products	1.5 (1.2–1.9)	1.9 (1.5–2.5)	—	—	2.0 (1.2–3.4)	—	1.5 (1.1–2.1)	2.3 (1.8–2.8)	—	**1.7 (1.4–2.1)**	**240,000**
Pipe tobacco	1.4 (1.1–1.7)	2.0 (1.6–2.6)	5.9 (3.3–10.5)	—	1.6 (1.0–2.5)	—	1.6 (1.3–2.1)	2.0 (1.6–2.6)	2.2 (1.4–3.4)	**1.7 (1.5–2.0)**	**260,000**
Any combustible tobacco product^§§^	10.4 (9.0–11.9)	13.6 (12.1–15.3)	20.9 (15.4–27.7)	4.4 (3.3–5.9)	13.0 (10.6–15.9)	—	12.7 (11.1–14.6)	11.2 (9.5–13.1)	15.3 (12.2–19.0)	**12.1 (10.8–13.4)**	**1,860,000**
Multiple tobacco products^¶¶^	9.3 (8.1–10.6)	12.4 (10.8–14.3)	22.6 (16.8–29.7)	3.0 (2.1–4.1)	9.4 (7.3–12.1)	—	12.1 (10.2–14.1)	10.6 (8.9–12.5)	12.9 (10.3–16.1)	**10.9 (9.6–12.3)**	**1,710,000**
**Middle school students (grades 6–8)**											
Any tobacco product^¶^	13.5 (11.4–15.9)	12.4 (10.9–14.0)	23.6 (18.1–30.1)	7.0 (4.4–10.9)	17.8 (15.2–20.7)	—	10.5 (8.4–13.1)	14.6 (13.0–16.3)	17.5 (13.4–22.6)	**12.9 (11.2–14.8)**	**1,530,000**
E-cigarettes	9.4 (7.8–11.2)	7.6 (6.6–8.8)	15.7 (11.0–21.8)	—	10.4 (8.5–12.7)	—	7.1 (5.6–9.0)	10.3 (9.1–11.6)	11.0 (8.1–14.8)	**8.5 (7.3–9.8)**	**1,000,000**
Cigarettes	3.7 (2.9–4.7)	3.6 (2.8–4.6)	5.5 (3.0–9.9)	—	4.5 (3.6–5.6)	—	3.2 (2.2–4.7)	4.1 (3.3–5.2)	5.2 (3.3–7.9)	**3.6 (2.9–4.5)**	**420,000**
Cigars^††^	1.9 (1.4–2.5)	2.1 (1.5–2.8)	4.8 (2.7–8.3)	—	3.2 (2.1–4.8)	—	1.4 (1.0–2.0)	2.5 (2.0–3.2)	—	**2.0 (1.6–2.5)**	**230,000**
Nicotine pouches	1.4 (1.1–1.9)	2.1 (1.7–2.7)	—	—	1.3 (0.8–2.2)	—	1.7 (1.2–2.3)	2.0 (1.5–2.6)	2.3 (1.5–3.7)	**1.8 (1.4–2.2)**	**190,000**
Other oral nicotine products^††^	2.2 (1.8–2.7)	2.2 (1.8–2.7)	—	—	1.9 (1.2–2.8)	—	1.8 (1.4–2.4)	2.9 (2.4–3.5)	3.1 (2.0–4.7)	**2.2 (1.8–2.6)**	**250,000**
Smokeless tobacco^††^	1.9 (1.4–2.6)	2.7 (2.1–3.5)	5.7 (3.7–8.7)	—	1.8 (1.1–2.7)	—	2.4 (1.7–3.5)	2.3 (1.7–2.9)	3.0 (1.8–4.9)	**2.3 (1.8–3.0)**	**260,000**
Hookahs	1.7 (1.3–2.2)	1.2 (0.9–1.6)	—	—	3.2 (2.4–4.4)	—	0.7 (0.4–1.0)	1.9 (1.4–2.5)	—	**1.4 (1.1–1.8)**	**160,000**
Heated tobacco products	1.2 (0.9–1.6)	1.4 (1.0–1.8)	—	—	1.6 (1.1–2.3)	—	0.8 (0.5–1.3)	1.8 (1.4–2.3)	—	**1.3 (1.0–1.6)**	**130,000**
Pipe tobacco	1.0 (0.8–1.4)	1.1 (0.8–1.5)	—	—	0.9 (0.5–1.5)	—	1.0 (0.7–1.3)	1.2 (0.9–1.7)	—	**1.1 (0.8–1.4)**	**120,000**
Any combustible tobacco product^§§^	5.9 (4.8–7.2)	5.9 (4.9–7.2)	10.9 (7.5–15.8)	3.6 (2.1–6.1)	9.3 (7.8–11.2)	—	4.7 (3.5–6.3)	6.5 (5.4–7.8)	8.0 (5.5–11.5)	**5.9 (4.9–7.1)**	**690,000**
Multiple tobacco products^¶¶^	5.3 (4.3–6.4)	5.6 (4.6–6.8)	10.6 (7.1–15.5)	3.4 (1.9–5.7)	6.5 (5.4–8.0)	—	4.7 (3.5–6.3)	6.3 (5.3–7.4)	6.4 (4.5–9.0)	**5.4 (4.5–6.5)**	**640,000**

**Abbreviations**: AI/AN = American Indian or Alaska Native; NH/PI = Native Hawaiian or Pacific Islander.

* Ever use is defined as ever having used the product, even once or twice. Because of missing data on the ever use questions, denominators for each tobacco product might differ. For each question, response options were “yes” or “no.”

^†^ Hispanic or Latino (Hispanic) persons might be of any race; all races listed are non-Hispanic.

^§^ Estimated weighted total number of students who reported ever use of tobacco product was rounded down to the nearest 10,000 persons. Overall estimates were reported based on 29,861 U.S. middle and high school students. School level was determined by reported grade level: high school = grades 9–12 (15,124) and middle school = grades 6–8 (14,554). The subgroup estimates might not sum to the overall population estimates because of rounding or exclusion of students who did not report sex, race and ethnicity, or grade level.

^¶^ Any tobacco product use is defined as ever use of one or more of the following tobacco products: e-cigarettes, nicotine pouches, cigarettes, cigars, smokeless tobacco (chewing tobacco, snuff, dip, or snus), other oral nicotine products, heated tobacco products, hookahs, pipe tobacco, or bidis (small, brown cigarettes wrapped in a leaf).

** Dashes indicate that data were statistically unreliable because of an unweighted denominator <50 or a relative SE >30%.

^††^ Cigars were defined as cigars, cigarillos, or little cigars. Smokeless tobacco was defined as chewing tobacco, snuff, dip, or snus. Other oral nicotine products were defined as lozenges, discs, tablets, gums, dissolvable tobacco products, and other products.

^§§^ Any combustible tobacco product use was defined as ever use of one or more of the following tobacco products: cigarettes, cigars, hookahs, pipe tobacco, or bidis.

^¶¶^ Multiple tobacco product use was defined as ever use of two or more of the following tobacco products: e-cigarettes, nicotine pouches, cigarettes, cigars, smokeless tobacco (chewing tobacco, snuff, dip, or snus), other oral nicotine products, heated tobacco products, hookahs, pipe tobacco, or bidis.

**TABLE 2:** Percentage of middle and high school students who reported current (previous 30-day) tobacco product use,* overall and by school level, product, sex, and race and ethnicity — National Youth Tobacco Survey, United States, 2024

Tobacco product	% (95% CI)									Total estimated no.^§^
	Sex		Race and ethnicity^†^						Total	
	Female	Male	AI/AN	Asian	Black or African American	White	Hispanic or Latino	Multiracial		
**Overall**										
Any tobacco product^¶^	7.7 (6.9–8.6)	8.5 (7.6–9.5)	16.3 (12.8–20.5)	3.3 (2.3–4.6)	10.0 (8.4–11.9)	7.8 (6.6–9.3)	8.4 (7.5–9.3)	9.0 (7.4–11.0)	**8.1 (7.4–8.9)**	**2,250,000**
E-cigarettes	6.1 (5.4–6.9)	5.8 (5.1–6.5)	11.5 (8.4–15.5)	2.3 (1.5–3.7)	7.0 (5.7–8.6)	5.9 (4.8–7.1)	6.1 (5.5–6.9)	6.6 (5.2–8.3)	**5.9 (5.3–6.6)**	**1,630,000**
Nicotine pouches	0.9 (0.7–1.1)	2.7 (2.2–3.2)	4.4 (2.8–7.0)	—**	1.0 (0.6–1.4)	2.2 (1.8–2.8)	1.7 (1.4–2.1)	1.4 (0.8–2.4)	**1.8 (1.5–2.1)**	**480,000**
Cigarettes	1.2 (1.0–1.5)	1.6 (1.3–2.0)	3.5 (2.1–5.6)	—	0.9 (0.6–1.4)	1.4 (1.2–1.8)	1.6 (1.3–2.0)	2.1 (1.3–3.5)	**1.4 (1.2–1.6)**	**380,000**
Cigars^††^	0.9 (0.7–1.2)	1.5 (1.2–1.9)	—	—	2.2 (1.5–3.1)	0.9 (0.7–1.2)	1.4 (1.1–1.8)	—	**1.2 (1.0–1.5)**	**330,000**
Smokeless tobacco^††^	0.7 (0.5–0.8)	1.7 (1.4–2.1)	3.6 (2.0–6.4)	—	0.8 (0.5–1.3)	1.3 (1.1–1.7)	1.3 (1.0–1.6)	1.3 (0.7–2.2)	**1.2 (1.0–1.4)**	**330,000**
Other oral nicotine products^††^	0.9 (0.7–1.1)	1.5 (1.2–1.8)	2.8 (1.6–5.0)	—	1.1 (0.7–1.6)	1.3 (1.0–1.6)	1.4 (1.1–1.6)	1.0 (0.6–1.6)	**1.2 (1.0–1.4)**	**320,000**
Heated tobacco products	0.7 (0.6–0.9)	0.9 (0.7–1.1)	—	—	0.9 (0.6–1.4)	0.6 (0.4–0.8)	1.2 (1.0–1.5)	0.8 (0.4–1.4)	**0.8 (0.7–1.0)**	**220,000**
Hookahs	0.8 (0.6–1.0)	0.7 (0.6–0.9)	—	—	1.5 (1.0–2.2)	0.4 (0.3–0.6)	0.9 (0.7–1.1)	0.8 (0.4–1.3)	**0.7 (0.6–0.9)**	**190,000**
Pipe tobacco	0.4 (0.3–0.6)	0.6 (0.4–0.7)	—	—	0.6 (0.3–1.0)	0.4 (0.3–0.5)	0.7 (0.5–0.9)	—	**0.5 (0.4–0.6)**	**130,000**
Any combustible tobacco product^§§^	2.4 (2.0–2.9)	3.1 (2.7–3.6)	6.3 (4.1–9.5)	—	4.1 (3.2–5.2)	2.4 (2.0–2.9)	2.9 (2.5–3.5)	3.9 (2.8–5.4)	**2.8 (2.5–3.2)**	**760,000**
Multiple tobacco products^¶¶^	2.5 (2.1–2.9)	3.6 (3.1–4.1)	6.9 (4.5–10.4)	1.1 (0.6–2.0)	3.3 (2.5–4.4)	3.0 (2.5–3.7)	3.1 (2.7–3.5)	3.8 (2.7–5.2)	**3.0 (2.7–3.4)**	**840,000**
**High school students (grades 9–12)**										
Any tobacco product^¶^	9.3 (8.2–10.5)	10.9 (9.5–12.5)	21.1 (15.7–27.8)	3.6 (2.5–5.2)	11.1 (8.8–13.9)	10.5 (8.6–12.6)	9.8 (8.4–11.3)	11.3 (8.9–14.3)	**10.1 (9.0–11.3)**	**1,580,000**
E-cigarettes	7.7 (6.7–8.9)	7.8 (6.7–9.0)	15.5 (10.5–22.1)	3.1 (2.0–4.9)	8.4 (6.6–10.7)	8.1 (6.6–10.0)	7.4 (6.4–8.5)	8.7 (6.6–11.4)	**7.8 (6.9–8.8)**	**1,210,000**
Nicotine pouches	0.8 (0.6–1.1)	3.9 (3.1–4.8)	5.6 (3.3–9.5)	—	0.9 (0.5–1.7)	3.3 (2.7–4.1)	2.0 (1.6–2.5)	—	**2.4 (2.0–2.9)**	**360,000**
Cigarettes	1.1 (0.9–1.5)	2.2 (1.8–2.7)	5.2 (3.0–8.7)	—	—	1.9 (1.4–2.4)	1.7 (1.4–2.2)	—	**1.7 (1.4–2.0)**	**250,000**
Cigars^††^	1.0 (0.7–1.4)	2.1 (1.6–2.7)	—	—	2.7 (1.7–4.2)	1.3 (0.9–1.7)	1.6 (1.2–2.2)	—	**1.5 (1.2–1.9)**	**230,000**
Smokeless tobacco^††^	0.6 (0.5–0.8)	2.3 (1.8–2.9)	—	—	—	1.8 (1.5–2.3)	1.4 (1.1–1.8)	—	**1.5 (1.2–1.8)**	**220,000**
Other oral nicotine products^††^	0.9 (0.7–1.2)	2.0 (1.6–2.4)	—	—	—	1.7 (1.3–2.2)	1.5 (1.2–1.8)	—	**1.4 (1.2–1.7)**	**210,000**
Heated tobacco products	0.7 (0.6–1.0)	1.0 (0.7–1.3)	—	—	—	0.7 (0.5–1.0)	1.3 (1.0–1.7)	—	**0.9 (0.7–1.1)**	**120,000**
Hookahs	0.7 (0.5–1.1)	0.9 (0.7–1.2)	—	—	1.6 (0.9–2.7)	0.4 (0.3–0.6)	1.0 (0.7–1.4)	—	**0.8 (0.6–1.1)**	**120,000**
Pipe tobacco	0.4 (0.2–0.6)	0.6 (0.4–0.8)	—	—	—	0.4 (0.2–0.6)	0.7 (0.5–1.0)	—	**0.5 (0.4–0.6)**	**70,000**
Any combustible tobacco product^§§^	2.6 (2.0–3.3)	4.0 (3.4–4.7)	8.0 (4.9–12.7)	—	4.4 (3.1–6.2)	3.0 (2.4–3.7)	3.3 (2.6–4.1)	5.0 (3.3–7.4)	**3.3 (2.9–3.9)**	**510,000**
Multiple tobacco products^¶¶^	2.7 (2.2–3.3)	4.7 (3.9–5.6)	9.3 (5.9–14.4)	—	3.6 (2.4–5.5)	4.1 (3.3–5.1)	3.2 (2.7–3.8)	4.6 (3.0–7.0)	**3.7 (3.2–4.3)**	**580,000**
**Middle school students (grades 6–8)**										
Any tobacco product^¶^	5.5 (4.5–6.7)	5.3 (4.5–6.3)	10.3 (6.9–15.2)	—	8.4 (6.8–10.3)	4.3 (3.2–5.6)	6.3 (5.4–7.3)	6.3 (4.6–8.6)	**5.4 (4.6–6.3)**	**640,000**
E-cigarettes	3.9 (3.1–4.9)	3.1 (2.5–3.8)	6.5 (3.7–11.1)	—	4.9 (3.8–6.4)	2.8 (2.0–3.9)	4.4 (3.7–5.3)	4.0 (2.6–6.1)	**3.5 (2.9–4.2)**	**410,000**
Nicotine pouches	0.9 (0.6–1.2)	1.1 (0.8–1.4)	—	—	0.9 (0.5–1.6)	0.8 (0.5–1.2)	1.2 (0.9–1.6)	—	**1.0 (0.8–1.2)**	**110,000**
Cigarettes	1.2 (0.9–1.6)	0.9 (0.6–1.4)	—	—	1.1 (0.6–1.8)	0.9 (0.6–1.4)	1.3 (1.0–1.8)	—	**1.1 (0.8–1.3)**	**120,000**
Cigars^††^	0.8 (0.5–1.1)	0.7 (0.4–1.2)	—	—	1.5 (0.9–2.4)	0.4 (0.3–0.8)	1.0 (0.7–1.3)	—	**0.8 (0.6–1.0)**	**80,000**
Smokeless tobacco^††^	0.7 (0.5–0.9)	1.0 (0.7–1.3)	—	—	_	0.7 (0.4–1.0)	1.0 (0.7–1.5)	—	**0.8 (0.6–1.1)**	**90,000**
Other oral nicotine products^††^	0.9 (0.7–1.2)	0.8 (0.6–1.1)	—	—	1.0 (0.6–1.7)	0.7 (0.4–1.0)	1.2 (0.9–1.7)	—	**0.9 (0.7–1.1)**	**100,000**
Heated tobacco products	0.7 (0.4–1.0)	0.8 (0.6–1.1)	—	—	0.9 (0.6–1.5)	—	1.1 (0.8–1.5)	—	**0.7 (0.6–1.0)**	**80,000**
Hookahs	0.7 (0.5–1.1)	0.4 (0.2–0.7)	—	—	1.3 (0.8–2.0)	—	0.6 (0.4–0.9)	—	**0.6 (0.4–0.8)**	**60,000**
Pipe tobacco	0.4 (0.3–0.7)	0.5 (0.3–0.8)	—	—	—	0.4 (0.2–0.6)	0.6 (0.4–1.0)	—	**0.5 (0.3–0.7)**	**50,000**
Any combustible tobacco product^§§^	2.1 (1.7–2.7)	2.0 (1.4–2.7)	—	—	3.5 (2.6–4.8)	1.5 (1.0–2.2)	2.3 (1.8–2.9)	2.5 (1.5–4.3)	**2.1 (1.6–2.6)**	**240,000**
Multiple tobacco products^¶¶^	2.1 (1.6–2.7)	2.0 (1.6–2.5)	—	—	2.7 (1.9–3.7)	1.6 (1.1–2.3)	2.6 (2.1–3.2)	2.7 (1.7–4.2)	**2.1 (1.7–2.5)**	**240,000**

**Abbreviation**: AI/AN = American Indian or Alaska Native.

* Current use is defined as use on ≥1 day during the previous 30 days for each product. Because of missing data on previous 30-day use questions, denominators for each tobacco product might differ.

^†^ Hispanic or Latino (Hispanic) persons might be of any race; all races listed are non-Hispanic. Estimates among non-Hispanic Native Hawaiian or Pacific Islander students, overall and by school level, were statistically unreliable for all measures and are not presented in this table.

^§^ Estimated weighted total number of students who reported current tobacco product use was rounded down to the nearest 10,000 persons. Overall estimates were reported based on 29,861 U.S. middle and high school students. School level was determined by reported grade level: high school = grades 9–12 (15,124) and middle school = grades 6–8 (14,554). The subgroup estimates might not sum to the overall population estimates because of rounding or exclusion of students who did not report sex, race and ethnicity, or grade level.

^¶^ Any tobacco product use is defined as current use of one or more of the following tobacco products on ≥1 day during the previous 30 days: e-cigarettes, nicotine pouches, cigarettes, cigars, smokeless tobacco (chewing tobacco, snuff, dip, or snus), other oral nicotine products, heated tobacco products, hookahs, pipe tobacco, or bidis (small, brown cigarettes wrapped in a leaf).

** Dashes indicate that data were statistically unreliable because of an unweighted denominator <50 or a relative SE >30%.

^††^ Cigars were defined as cigars, cigarillos, or little cigars. Smokeless tobacco was defined as chewing tobacco, snuff, dip, or snus. Other oral nicotine products were defined as lozenges, discs, tablets, gums, dissolvable tobacco products, and other products.

^§§^ Any combustible tobacco product use was defined as current use of one or more of the following tobacco products: cigarettes, cigars, hookahs, pipe tobacco, or bidis.

^¶¶^ Multiple tobacco product use was defined as current use of two or more of the following tobacco products: e-cigarettes, nicotine pouches, cigarettes, cigars, smokeless tobacco (chewing tobacco, snuff, dip, or snus), other oral nicotine products, heated tobacco products, hookahs, pipe tobacco, or bidis.

### Types of Tobacco Products Used

E-cigarettes were the most commonly reported currently used tobacco product (5.9%) among all students, followed by nicotine pouches (1.8%), cigarettes (1.4%), cigars (1.2%), smokeless tobacco (1.2%), other oral nicotine products (1.2%), heated tobacco products (0.8%), hookahs (0.7%), and pipe tobacco (0.5%). Among students who had ever used e-cigarettes, 43.6% reported current e-cigarette use.

### High School Student Tobacco Product Use

Among high school students, 10.1% reported current use of any tobacco product, 3.3% reported current use of any combustible tobacco product (32.7% of those who reported current use of any tobacco product), and 3.7% reported current use of multiple tobacco products (36.6% of those who reported any tobacco product use). E-cigarettes were the most commonly used product (7.8%), followed by nicotine pouches (2.4%), cigarettes (1.7%), cigars (1.5%), smokeless tobacco (1.5%), other oral nicotine products (1.4%), heated tobacco products (0.9%), hookahs (0.8%), and pipe tobacco (0.5%).

### Middle School Student Tobacco Product Use

Among middle school students, 5.4% reported current use of any tobacco product, 2.1% reported current use of any combustible tobacco product (38.9% of those who reported current use of any tobacco product), and 2.1% reported current use of multiple tobacco products (38.9% of those who reported any tobacco product use). E-cigarettes were the most commonly used product (3.5%), followed by cigarettes (1.1%), nicotine pouches (1.0%), other oral nicotine products (0.9%), cigars (0.8%), smokeless tobacco (0.8%), heated tobacco products (0.7%), hookahs (0.6%), and pipe tobacco (0.5%).

### Trends in Tobacco Product Use Prevalence Among Middle and High School Students

From 2023 (*3*) to 2024, statistically significant declines occurred among all students in current use of any tobacco product (from 10.0% to 8.1%), e-cigarettes (from 7.7% to 5.9%) (*2*), and hookahs (from 1.1% to 0.7%) (Figure). Among high school students, declines occurred in current use of any tobacco product (from 12.6% to 10.1%) and e-cigarettes (from 10.0% to 7.8%). Among middle school students, no significant change in current use of any individual tobacco product or composite tobacco product use measure was observed. During 2023–2024, whereas any tobacco product use prevalence declined among female students (from 11.2% to 7.7%) and Hispanic students (from 11.7% to 8.4%), use increased among AI/AN students (from 8.0% to 16.3%). By product type, from 2023 to 2024, declines occurred among female students in current use of e-cigarettes (from 9.3% to 6.1%) and multiple tobacco products (from 3.4% to 2.5%) and among Hispanic students in current use of e-cigarettes (from 8.5% to 6.1%), cigars (from 2.2% to 1.4%), hookahs (from 1.3% to 0.9%), and multiple tobacco products (from 3.9% to 3.1%). In contrast, increases occurred among White students in current use of nicotine pouches (from 1.4% to 2.2%) and among AI/AN students in current use of e-cigarettes (from 5.9% to 11.5%), other oral nicotine products (from 0.5% to 2.8%), any combustible tobacco product (from 2.7% to 6.3%), and multiple tobacco products (from 2.0% to 6.9%). No significant changes occurred in current use of cigarettes, smokeless tobacco, heated tobacco products, or pipe tobacco among all racial and ethnic groups.

**FIGURE:**
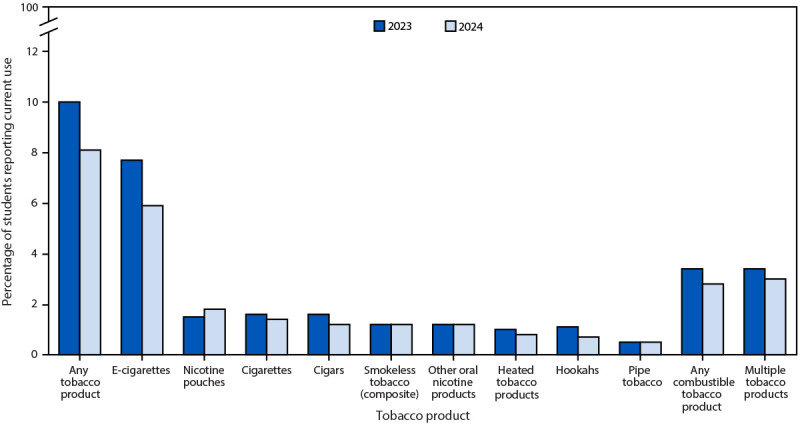
Current use of selected tobacco products,* any tobacco product,^†^ any combustible tobacco product,^§^ and multiple tobacco products^¶^ by middle and high school students — National Youth Tobacco Survey, United States, 2023 and 2024** * Current use is defined as use on ≥1 day during the past 30 days for each product. ^†^ Any tobacco product use is defined as current use of one or more of the following tobacco products on ≥1 day during the past 30 days: e-cigarettes, nicotine pouches, cigarettes, cigars (cigars, cigarillos, or little cigars), smokeless tobacco (composite [chewing tobacco, snuff, dip, or snus]), other oral nicotine products, heated tobacco products, hookahs, pipe tobacco, or bidis (small, brown cigarettes wrapped in a leaf). ^§^ Any combustible tobacco product use was defined as current use of one or more of the following tobacco products: cigarettes, cigars, hookah, pipe tobacco, or bidis. ^¶^ Multiple tobacco product use was defined as current use of two or more of the following tobacco products: e-cigarettes, nicotine pouches, cigarettes, cigars, smokeless tobacco (composite [chewing tobacco, snuff, dip, or snus]), other oral nicotine products, heated tobacco products, hookahs, pipe tobacco, or bidis. ** During 2023–2024, statistically significant declines in the use of any tobacco product, e-cigarettes, and hookahs were observed. No statistically significant change in use of nicotine pouches, cigarettes, cigars, smokeless tobacco, other oral nicotine products, heated tobacco products, pipe tobacco, any combustible tobacco, or multiple tobacco products occurred.

## Discussion

During 2023–2024, among all middle school and high school students, current use of any tobacco product declined by an estimated 550,000 students, largely driven by the decline in high school e-cigarette use (from 1.56 million to 1.21 million) (*3*), and reaching the lowest level ever measured by NYTS. Despite these declines, approximately one in 12 middle and high school students reported current use of any tobacco product during 2024, including approximately one in 10 high school students and approximately 1 in 20 middle school students. Approximately two in five students who had ever used a tobacco product currently used them.

Since 2014, e-cigarettes have been the most used tobacco product among U.S. youths (*4*). From 2023 (*3*) to 2024, current use of e-cigarettes declined significantly among high school students. The decline in high school student e-cigarette use is likely attributable to multiple factors, including ongoing activities at the national, state, and local levels to implement tobacco control strategies. A similar decline in e-cigarette use among high school students occurred from 2022 to 2023 (*3*). E-cigarette use did not change among middle school students from 2023 to 2024, similar to use from 2022 to 2023 (*3*).

In 2024, 1.7% of high school students and 1.1% of middle school students reported current cigarette smoking, the lowest prevalence ever recorded by NYTS. However, youths continue to use other tobacco products, including e-cigarettes and nicotine pouches (*2*). As the tobacco product market continues to evolve, vigilant monitoring of emerging tobacco product trends among youths is important. Nicotine pouch sales have substantially increased nationwide since 2016^§§§^ (*5*); although sales data do not indicate which age groups are using the products, NYTS data indicate use of nicotine pouches among youths remains relatively low (*2*). However, for the first time, nicotine pouches were the second most common currently used tobacco product (1.8%); nearly one million (890,000) students reported ever using nicotine pouches in 2024. CDC and FDA will continue monitoring tobacco product use among youths, especially e-cigarette and nicotine pouches, and address any potential increase in use of these products (*2*,*5*).

Current use of any tobacco product was similar among male and female students; however, males were more likely to report current use of multiple tobacco products. Consistent with previous reports (*6*), among all racial and ethnic groups, AI/AN students reported the highest prevalence of current use of any tobacco product, of e-cigarettes, and of multiple tobacco products. Further, during 2023–2024, whereas any tobacco product use declined for Hispanic students and remained stable for all other racial and ethnic groups, it increased among AI/AN students, highlighting disparities in tobacco product use. Activities aimed at reducing disparities are a critical part of tobacco prevention and control measures (*3*).

### Limitations

The findings in this report are subject to at least five limitations. First, data were obtained by self-report, which is subject to social desirability and recall biases, although previous research suggests that self-reported measures of tobacco use among persons aged 12–21 years correlate with tobacco use biomarkers (*7*). Second, these findings might not be generalizable to youths who are home-schooled, have dropped out of school, are in detention centers, or are enrolled in alternative schools. Third, the 2023 estimate for nicotine pouch use among middle school students was suppressed; therefore, it could not be compared with the 2024 estimate. Fourth, some AI/AN populations use traditional tobacco in cultural ceremonies of medicinal and spiritual importance (*8*). NYTS does not distinguish between use of ceremonial and commercial tobacco use; therefore, estimates among AI/AN youth might also include ceremonial tobacco use. Finally, because of small sample sizes, many estimates for racial and ethnic population groups were not reliable, particularly for less prevalent tobacco products and among the Non-Hispanic Native Hawaiian or Pacific Islander population.

### Implications for Public Health Practice

In 2024, 8.1% (2.25 million) of U.S. middle and high school students reported current tobacco product use. From 2023 to 2024, substantial declines in current use of any tobacco product and e-cigarettes among high school students occurred; however, no change was observed among middle school students. Multiple factors continue to influence tobacco product use and initiation among adolescents including availability of youth-appealing flavored products, marketing, harm misperceptions, the emergence of new flavor types (e.g., ice flavors [flavors that combine cooling and fruit or sweet flavors, such as blueberry ice or strawberry ice]), and product features (*3*). Given the negative health consequences of tobacco use (*9*) and the unique harms associated with adolescent nicotine exposure (*1*), prevention of youth tobacco product use is crucial. Preventing initiation and promoting cessation require a comprehensive approach at the local, state, and national levels. Evidence-based tobacco prevention strategies include price increases, mass media campaigns to educate youths about the harmful effects of all tobacco products, and implementation of comprehensive smoke-free policies that include e-cigarettes (*1*).

## Footnotes

* The term “tobacco” as used in this report refers to commercial tobacco products and not to the sacred and traditional use of tobacco by some American Indian communities; however, NYTS does not distinguish between use of ceremonial and commercial use.

† Data were weighted to account for complex survey design and to adjust for nonresponse. The weighted proportions of students in each grade matched national population proportions for U.S. public and private schools using combined data from the National Center for Education Statistics (the 2021–2022 Common Core of Data and the 2019–2020 Private School Universe Survey) and spring 2023 data from Market Data Retrieval Inc. Population total estimates were rounded down to the nearest 10,000 persons.

§ Products include e-cigarettes, nicotine pouches, cigarettes, cigars (cigars, cigarillos, or little cigars), smokeless tobacco (chewing tobacco, snuff, dip, or snus), other oral nicotine products (lozenges, discs, tablets, gums, dissolvable tobacco products, and other products), heated tobacco products, hookahs, and pipe tobacco. Consistent with 2023 NYTS reporting analyses, dissolvable tobacco products were classified as other oral nicotine products.

¶ Small, flavored pouches that contain nicotine. Users place them in their mouth between the lip and gum. Unlike other smokeless tobacco products such as snus, dip, or chewing tobacco, nicotine pouches do not contain any tobacco leaf.

** Heated tobacco products include heat-processed tobacco leaf in the form of sticks (“heatsticks”), plugs, or capsules that produce a vapor the user inhales. This is different from e-cigarettes, which heat a liquid to produce a vapor.

†† Any tobacco product use was defined as the use of one or more of the following tobacco products: e-cigarettes, nicotine pouches, cigarettes, cigars, smokeless tobacco (chewing tobacco, snuff, dip, or snus), other oral nicotine products, heated tobacco products, hookahs, pipe tobacco, or bidis (small, brown cigarettes wrapped in a leaf).

§§ Any combustible tobacco product use was defined as the use of one or more of the following tobacco products: cigarettes, cigars, hookahs, pipe tobacco, or bidis.

¶¶ Multiple tobacco product use was defined as the use of two or more of the following tobacco products: e-cigarettes, nicotine pouches, cigarettes, cigars, smokeless tobacco (chewing tobacco, snuff, dip, or snus), other oral nicotine products, heated tobacco products, hookahs, pipe tobacco, or bidis.

*** 45 C.F.R. part 46.102(l)(2), 21 C.F.R. part 56; 42 U.S.C. Sect. 241(d); 5 U.S.C. Sect. 552a; 44 U.S.C. Sect. 3501 et seq.

††† Estimates for non-Hispanic Native Hawaiian or Pacific Islander students, overall and by school level, were statistically unreliable for all current use measures and are not reported.

§§§ The questions about nicotine pouch use were first added to NYTS in 2021.
